# Complete Genome Sequence of Systemically Disseminated Sequence Type 8 Staphylococcal Cassette Chromosome *mec* Type IVl Community-Acquired Methicillin-Resistant *Staphylococcus aureus*

**DOI:** 10.1128/genomeA.00852-17

**Published:** 2017-08-31

**Authors:** Junzo Hisatsune, Hideharu Hagiya, Sumiko Shiota, Motoyuki Sugai

**Affiliations:** a Department of Bacteriology, Hiroshima University Graduate School of Biomedical & Health Sciences, Hiroshima, Japan; b Project Research Center for Nosocomial Infectious Diseases, Hiroshima University, Hiroshima, Japan; c Department of General Medicine, Okayama University Graduate School of Medicine, Dentistry and Pharmaceutical Sciences, Okayama, Japan; d Department of Pathogenic Microbiology, School of Pharmacy, Shujitsu University, Okayama, Japan

## Abstract

*Staphylococcus aureus* JH4899, a community-acquired methicillin-resistant *Staphylococcus aureus* (CA-MRSA) isolate collected from a patient with systematically disseminated infection, is classified as sequence type 8 and carries the staphylococcal cassette chromosome *mec* type IVl (SCC*mec*IVl). It produces TSST-1, SEC, a newly discovered enterotoxin (SE1), and epidermal cell differentiation inhibitor A (EDIN-A). Here, we present the complete genome sequence of the chromosome and a plasmid harboring the *se1* and *ednA* genes.

## GENOME ANNOUNCEMENT

Community-acquired methicillin-resistant *Staphylococcus aureus* (CA-MRSA) is genetically heterogeneous, and several genotypes are spreading worldwide. Recently, emergence of the Japanese CA-MRSA strain (CA-MRSA/J), which is sequence type 8 (ST8) of multiple locus sequencing typing (MLST), has been reported (1). Genotypical CA-MRSA/J is a lineage divergent from ST8 CA-MRSA, USA300, which is predominant in the United States (2).

We previously reported a patient with systematically disseminated CA-MRSA/J infection and described the genotypic analysis (3). Here, we report the complete genome sequence of CA-MRSA/J JH4899, which carries the staphylococcal cassette chromosome *mec* type IVl (SCC*mec*IVl) isolated in 2012 from the expectorated sputum of the patient.

The genomic DNA was isolated from JH4899 grown on tryptic soy broth (TSB) at 37°C using the QIAamp DNA minikit (Qiagen, USA) with the addition of lysostaphin (10 µg/mL) and incubation at 37°C for 1 h. The DNA library was prepared using the Nextera XT DNA library prep kit (Illumina, Inc., San Diego, CA) and sequenced as paired-end reads using an Illumina MiSeq platform and MiSeq reagent kit v2 (500 cycles) (Illumina). The run generated 1,552,210 reads (approximately 123-fold genome coverage). Illumina reads were assembled *de novo* using CLC Genomics Workbench version 7 (CLC bio, Inc., Aarhus, Denmark). These analyses yielded 5 scaffolds and 38 contigs, and gaps were closed by sequencing PCR products spanning the gaps. The completed genome sequence was automatically annotated using the Microbial Genome Annotation Pipeline (MiGAP) (4) and manually corrected using *in silico* Molecular Cloning Genomics Edition software (In Silico Biology, Inc, Yokohama, Japan) (5).

The JH4899 genome comprised a 2,808,921-bp chromosome and 32,580-bp plasmid (pJSA01), with average G+C contents of 32.8% and 28.7%, respectively. The chromosome contained 2,619 predicted protein-coding sequences (CDSs), 59 tRNA genes, and 6 rRNA operons. pJSA01 encoded 45 predicted CDSs.

The SCC*mec*IVl of JH4899 contained a 3,939-bp *sasL* gene encoding a cell wall-anchored surface protein, SasL (6). Chromosomal DNA included the pathogenicity island SaPIj50, carrying the toxic shock syndrome toxin gene (*tst-1*), enterotoxin C (*sec*), and enterotoxin L (*sel*) genes, and phage ϕSa3j, carrying *sak*, *scn*, and *sep*, encoding staphylokinase, staphylococcal complement inhibitor, and enterotoxin P, respectively (1). The JH4899 chromosomal genome carried aminoglycoside [*aac*(*6′*)/*aph*(*2′′*)] resistance transposon Tn*4001*, and transposon Tn*552*, carrying the β-lactam resistance gene (*blaZ*). Like NN50, the first CA-MRSA/J isolated in Japan, the JH4899 genome did not carry genes for Panton-Valentine leukocidin (PVL) or an arginine catabolic mobile element (ACME), which are present in the prophage and SCC*mec*IVa on the USA300 genome, respectively (2). Comparison of the JH4899 complete genome sequence with the draft genome sequence of NN50 indicated that their genome sequences were almost identical except that JH4899 possessed the plasmid pJSA01 but NN50 did not (1).

pJSA01 carried two virulence genes encoding a newly discovered enterotoxin (SE1) and ADP-ribosyltransferase and epidermal cell differentiation inhibitor A (EDIN-A) (7). These genes were tandemly sandwiched by three IS*257*s embedded in the plasmid. Furthermore, this plasmid possessed an antiseptic efflux protein gene, *qacB*.

The complete genome sequence of JH4899 will contribute to further understanding of the virulence mechanism of CA-MRSA/J infection.

### Accession number(s).

Whole-genome sequences of *S. aureus* JH4899 and pJSA01 have been deposited in the DDBJ/EMBL/GenBank under the accession numbers AP014921 and AP014922 for the chromosome and plasmid, respectively. The version described in this paper is the first version.
